# Multi-Serotype Pneumococcal Nasopharyngeal Carriage Prevalence in Vaccine Naïve Nepalese Children, Assessed Using Molecular Serotyping

**DOI:** 10.1371/journal.pone.0114286

**Published:** 2015-02-02

**Authors:** Rama Kandasamy, Meeru Gurung, Anushil Thapa, Susan Ndimah, Neelam Adhikari, David R. Murdoch, Dominic F. Kelly, Denise E. Waldron, Katherine A. Gould, Stephen Thorson, Shrijana Shrestha, Jason Hinds, Andrew J. Pollard

**Affiliations:** 1 Oxford Vaccine Group, Department of Paediatrics, University of Oxford, Oxford, United Kingdom; 2 NIHR Oxford Biomedical Research Centre, Oxford, United Kingdom; 3 Paediatric Research Unit, Patan Academy of Health Sciences, Kathmandu, Nepal; 4 Department of Pathology, University of Otago, Christchurch, New Zealand; 5 Division of Clinical Sciences, St. George’s, University of London, London, United Kingdom; Centers for Disease Control & Prevention, UNITED STATES

## Abstract

Invasive pneumococcal disease is one of the major causes of death in young children in resource poor countries. Nasopharyngeal carriage studies provide insight into the local prevalence of circulating pneumococcal serotypes. There are very few data on the concurrent carriage of multiple pneumococcal serotypes. This study aimed to identify the prevalence and serotype distribution of pneumococci carried in the nasopharynx of young healthy Nepalese children prior to the introduction of a pneumococcal conjugate vaccine using a microarray-based molecular serotyping method capable of detecting multi-serotype carriage. We conducted a cross-sectional study of healthy children aged 6 weeks to 24 months from the Kathmandu Valley, Nepal between May and October 2012. Nasopharyngeal swabs were frozen and subsequently plated on selective culture media. DNA extracts of plate sweeps of pneumococcal colonies from these cultures were analysed using a molecular serotyping microarray capable of detecting relative abundance of multiple pneumococcal serotypes. 600 children were enrolled into the study: 199 aged 6 weeks to <6 months, 202 aged 6 months to < 12 months, and 199 aged 12 month to 24 months. Typeable pneumococci were identified in 297/600 (49·5%) of samples with more than one serotype being found in 67/297 (20·2%) of these samples. The serotypes covered by the thirteen-valent pneumococcal conjugate vaccine were identified in 44·4% of samples containing typeable pneumococci. Application of a molecular serotyping approach to identification of multiple pneumococcal carriage demonstrates a substantial prevalence of co-colonisation. Continued surveillance utilising this approach following the introduction of routine use of pneumococcal conjugate vaccinates in infants will provide a more accurate understanding of vaccine efficacy against carriage and a better understanding of the dynamics of subsequent serotype and genotype replacement.

## Introduction

Disease due to pneumococcus is responsible for 11% of all deaths in children less than five years of age worldwide, with a disproportionate number of these deaths in developing countries [1]. Pneumonia is responsible for the largest burden of disease however pneumococcal meningitis has a higher mortality and for children who do survive meningitis a significant proportion develop long-term sequelae [2]. Previous work has demonstrated the importance of both pneumonia and meningitis due to pneumococcus in Nepalese children in Kathmandu [3].

Nasopharyngeal colonisation with pneumococcus is a necessary precursor to invasive and mucosal disease [4, 5]. Children in resource poor countries have a higher prevalence of pneumococcal colonisation from an earlier age than children from other countries, which has been attributed to factors such as poor nutrition [6], and crowded housing conditions [7]. Conventional cross-sectional studies of pneumococcal carriage typically detect the presence of a single serotype per participant. Such studies reinforce an overly simplified perception of the biology of pneumococcal carriage. Both serotype and genetic diversity in organisms occupying a limited niche indicate the potential for complex interactions between pneumococci and other species within an individual. It has already been shown that numerous pneumococcal serotypes may colonise a child over time [7–9]. Furthermore it is increasingly recognised that concurrent colonisation with more than one serotype of pneumococcus occurs, and in some settings at high frequency [7, 10–12]. Studies looking at the overall prevalence of multiple pneumococcal serotype carriage, provide a wide range of estimates from 1·5% to 40% [10–15]. The precise quantification of multiple serotype carriage is essential to the understanding of interactions, such as gene exchange between serotypes [16, 17], how this ecology is disturbed by immunisation, and improving the accuracy of models utilising carriage prevalence to predict vaccine impact on disease [4, 18].

The World Health Organisation (WHO) has highlighted the importance of developing more sensitive techniques for measuring carriage of multiple pneumococcal serotypes [15]. There are now nearly 100 different known serotypes of pneumococci. Conventional serotyping has its limitations, in that it is labour intensive and is usually undertaken on single colonies from culture. Determination of pneumococcal serotype using recently developed microarray techniques for the analysis of genetic material from microbial cultures has the advantage of being able to identify multiple serotypes within a single sample, to quantify relative abundance, and to determine the presence of antibiotic resistance genes [19].

Pneumococcal conjugate vaccines are extremely effective in reducing invasive disease through a combination of inducing antibody that mediates direct protection and reducing carriage that gives herd immunity [20–22]. The thirteen valent (serotypes 1, 3, 4, 5, 6A, 6B, 7F, 9V, 14, 18C, 19A, 19F, and 23F) conjugate vaccine (PCV13) is now used in routine infant immunisation schedules in many industrialised countries. The introduction of routine immunisaiton with pneumococcal conjugate vaccines (PCVs) in non-industrialised countries has occurred more slowly. This has been due to a number of factors including a lack of funding, and the scarcity of data on disease burden and causative serotypes, which varies geographically. In Nepal there is currently no pneumococcal conjugate vaccine used in routine infant immunisation however there has been approval for New Vaccine Support of PCV13 by the Global Alliance for Vaccines and Immunisation (GAVI) [23], which will allow vaccine introduction in the near future.

This study was undertaken to describe the prevalence and serotype distribution of pneumococcal carriage among healthy Nepalese children below two years of age in Kathmandu prior to the introduction of a pneumococcal conjugate vaccine, utilising a molecular serotyping technique, enabling detection of multiple serotype carriage.

## Materials and Methods

### Study Design

A cross-sectional study involving healthy children was conducted in Patan Hospital, Kathmandu, Nepal from May to October 2012. The primary objective was to determine the overall serotype distribution among healthy Nepalese children less than 2 years of age. The secondary objectives were to assess prevalence of multi-serotype carriage, the vaccine/non-vaccine serotype specific carriage prevalence and age specific carriage prevalence of serotypes in Nepalese children.

### Ethics Statement

Ethical approval was obtained from the Oxford Tropical Research Ethics Committee (OXTREC 17–12) and the Nepal Health Research Council (Reg. No. 31/2012). Parents/guardians of eligible infants provided informed written consent on behalf of their children.

### Study Participants

Equal numbers of participants were to be recruited from each of three age groups: 6 weeks to 5 months + 29 days, 6 months to 11 months + 29 days and 12 months to 24 months. In a prior study of pneumococcal carriage in Nepal using conventional microbiological methods over 40 serotypes were detected with the maximum prevalence of a single serotype being 8·5% [24]. In that study the sample size of 600 was based on the feasibility of recruitment logistics as well as the precision of the results obtained. In this study a total sample size of 600 was selected to give a similar precision for detection of serotype prevalence. Parents/guardians of healthy children and their siblings who were attending Patan Hospital for routine check-up, vaccination, or visiting patients as well as parents/guardians of children with minor injuries, were approached for consent and enrolment into the study.

Participants were excluded if they had documented evidence of previous pneumococcal vaccination, recent antibiotic treatment, or had a febrile illness/temperature of more than 38ºC. Due to the higher prevalence of nasopharyngeal carriage observed in rural compared to urban populations in Nepal [24], only children who resided within the urbanised setting of the Kathmandu Valley were included. Demographic and medical data were collected and participants swabbed by trained research staff according to WHO guidelines [15].

### Specimen Collection, Transport and Microbiology

A single nasopharyngeal nichrome swab (MWE medical wire, Wiltshire, England) was obtained from each participant, placed in skim-milk-tryptone-glucose-glycerin (STGG) medium and stored at -70ºC until transportation on dry ice to the Oxford Vaccine Group, UK. Following defrosting, the nasopharyngeal swabs were vortexed and three dilutions (neat, 1:10, 1:100) of STGG medium sub-cultured on selective colistin oxolinic blood agar + 5% horse blood plates (Oxoid Ltd, UK) at 37ºC and 5%CO_2_ overnight. Culture plates for the preparation of presumptive pneumococcus samples were selected based on high density of morphologically distinct colonies and a suspension of plate sweep (a swab is passed in a uniform pattern back and forth across the entire culture plate) frozen for analysis by the Bacterial Microarray Group at St. George’s, University of London (BµG@S).

### DNA extraction and Microarray Analysis

Genomic DNA was extracted from presumptive pneumococcal suspensions using QIAamp DNA Mini Kit protocol (Qiagen, Germany). Briefly; the suspension was centrifuged to pellet the bacterial, re-suspended in 180µL freshly prepared lysis buffer (20mg/mL lysozyme, 1mM Tris-HCL, 500mM EDTA, 10% Triton) and incubated at 37ºC for 60 min. Proteinase K (20µL) and Qiagen buffer AL (200µL) were added to the mixture and incubated for another 60 min at 56ºC. 4µL RNase A were added and incubated at room temperature for 5 min before incubation at 70ºC for 10 min. The standard manufacturer’s extraction protocol was followed after lysis and the DNA extracted was stored at 4ºC for microarray analysis.

Molecular serotyping was performed using the BµG@S SP-CPSv1.4.0 microarray [19]. Briefly, DNA samples were fluorescently labelled and hybridized to the Agilent 8×15K format microarray according to manufacturer’s instructions for the Agilent genomic DNA ULS labelling and oligo aCGH hybridisation reagent kits. Microarray data was statistically analysed using a Bayesian hierarchical model [25] to determine the serotype, or combination of serotypes, present in the sample and assign a relative abundance of each serotype detected. Additional components of the microarray enabled detection of antibiotic resistance genes and assessed genetic relatedness of samples by arrayCGH.

### Statistical Analysis

The Binomial Exact method was used to generate 95% confidence intervals of proportions. Demographic and antibiotic resistance data were analysed using a Chi-square analysis of 3 by 2 contingency tables. Associations between co-colonisers, and primary and non-primary frequency data for each serotype, which was isolated on 5 or more occasions, were analysed using the two-tailed Binomial test, where the observed proportion of primary to non-primary isolates for each serotype were compared to the overall proportion of primary (0.72) to non-primary (0.28) isolates. All analyses were performed in Graphpad Prism 6.

## Results

### Enrolment and Demographic Characteristics

A total of 600 children from the Kathmandu Valley, Nepal were enrolled in the study from May to October 2012. There were no significant differences in demographic characteristics between the age groups except for the expected increase in immunisation coverage with age (Table 1).

**Table 1:** Characteristics of Nepal Pneumococcal Carriage Study Subjects, n (%).

		**6wk-<6mo**	**6mo-<12mo**	**12mo-24mo**	
**Age (median, IQR)**		2·7 (2·4–3·6) months	9·18 (7·7–9·5) months	15·0 (13·2–18·6) months	
**Sex**	Male	119 (59·8)	113 (55·9)	104 (52·3)	p = 0·32
	Female	80 (40·2)	89 (44·1)	95 (47·7)	
**Vaccination**	BCG	197 (99)	196 (97)	195 (98)	
	HepB/Hib/DTP/OPV (1 dose)	169 (84·9)	190 (94)	188 (94·5)	
	(2 doses)	135 (67·8)	185 (91·6)	176 (88·4)	
	(3 doses)	73 (36·7)	182 (90·1)	175 (87·9)	
	Measles (at least 1 dose)	0 (0)	136 (67·3)	188 (94·5)	
**Neonatal History**	Term	192 (96·5)	197 (97·5)	190 (95·5)	p = 0·49
	Preterm	6 (3)	4 (2)	8 (4)	
	Birth weight (median)	3·04 kg	3·02 kg	3·01 kg	

### Comparison of Serotype Specific Pneumococcal Carriage Prevalence Between Age Groups

**Fig 1:**
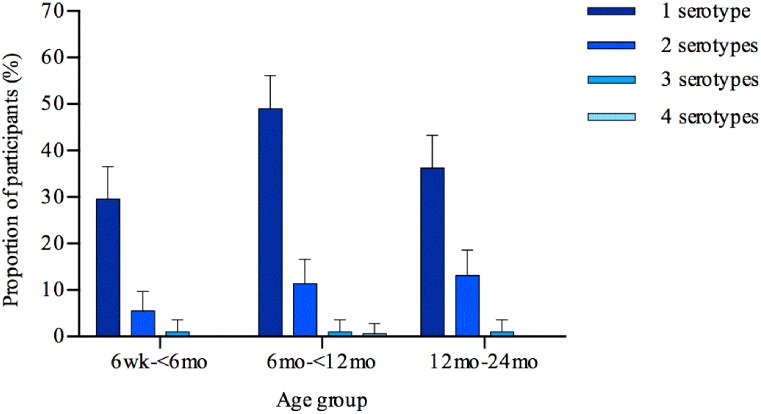
Multiple pneumococcal carriage by age group. The proportion of nasopharyngeal swabs from children from the Kathmandu Valley, Nepal, positive for pneumococcal serotype/s by microarray analysis categorised by age group. Error bars indicate 95% confidence interval upper limits.

Of the 600 cultures from swabs, 375 yielded presumptive pneumococci from which plate sweeps were collected and processed for microarray analysis. A total of 309/600 (51·5%) samples had pneumococcus identified on microarray. Typeable pneumococci were detected in 297/309 (96·1%) with 60/297 (20·2%) having multiple serotypes identified. By age group swabs yielded positive results for pneumococcal serotypes in 36·2% (95% CI 30–43%), 61·9% (95% CI 55–69%), and 50·2% (95% CI 43–57%) of the 6 week to < 6 months, 6 months to < 12 months and 12 to 24 months age groups respectively (p<0·0001, Fig. 1). For each of the three corresponding age groups, the number of pneumococcal serotypes identified on each swab were then categorised according to whether there were one (29·7%, 49% and 36·2%), two (5·5%, 11·4%, and 13·1%), three (1%, 1%, and 1%), or four (0%, 0·5% and 0%) serotypes found.

### Presence of Pneumococcal Vaccine Serotypes

**Fig 2:**
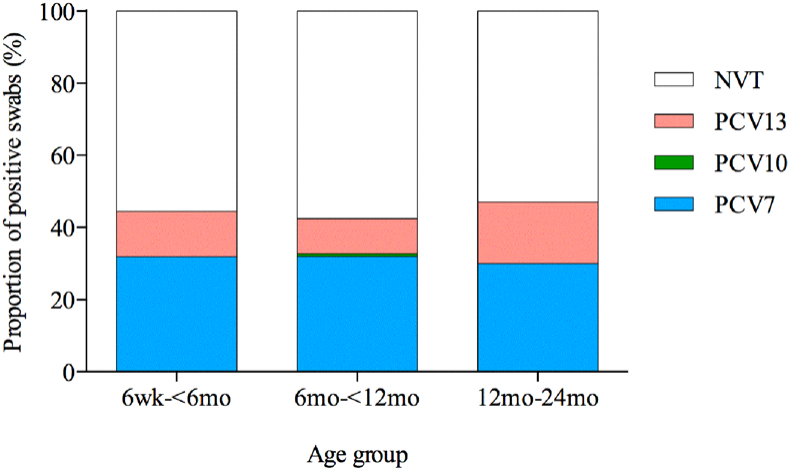
Nasopharyngeal carriage of pneumococcal conjugate vaccine serotypes. The proportion of nasopharyngeal swabs from children from the Kathmandu Valley, Nepal, that had at least one pneumococcal serotype contained within each of the pneumococcal conjugate vaccines (PCV7, PCV 10, and PCV 13) for each age group. Those swabs that did not contain any of the vaccine serotypes were classified as non-vaccine types (NVT).

Swabs that were identified as having a pneumococcal serotype contained within PCV7 (serotypes 4, 6B, 9V, 14, 18C, 19F, and 23F), PCV10 (additional serotypes 1, 5, 7F) and PCV13 were categorized for each age group (Fig. 2). Overall 132/297 (44·4%) of the pneumococcal serotype positive swabs had at least one PCV13 serotype present (44·4%, 42·4%, and 47% in the 6 week to < 6 months, 6 months to < 12 months and 12 to 24 months age groups respectively). Of the PCV7 serotypes 31·9%, 32%, and 30% of the pneumococcal serotype positive swabs were found to contain at least one vaccine serotype in the 6 week to < 6 months, 6 months to < 12 months and 12 to 24 months age groups respectively. In the 6 week to < 6 months, and 12 to 24 months age groups there was no additional effect conferred by PCV10 compared to PCV7. A 0·8% additional coverage was offered by PCV10 over PCV7 in the 6 months to < 12 months age group. Swabs that only contained serotypes not covered by PCV13 (NVT) accounted for 55·6%, 57·6%, and 53% in the 6 week to < 6 months, 6 months to < 12 months and 12 to 24 months age groups respectively (p = 0·20).

### Presence of Non-typeable and Closely Related *Streptococcus* spp.

**Fig 3:**
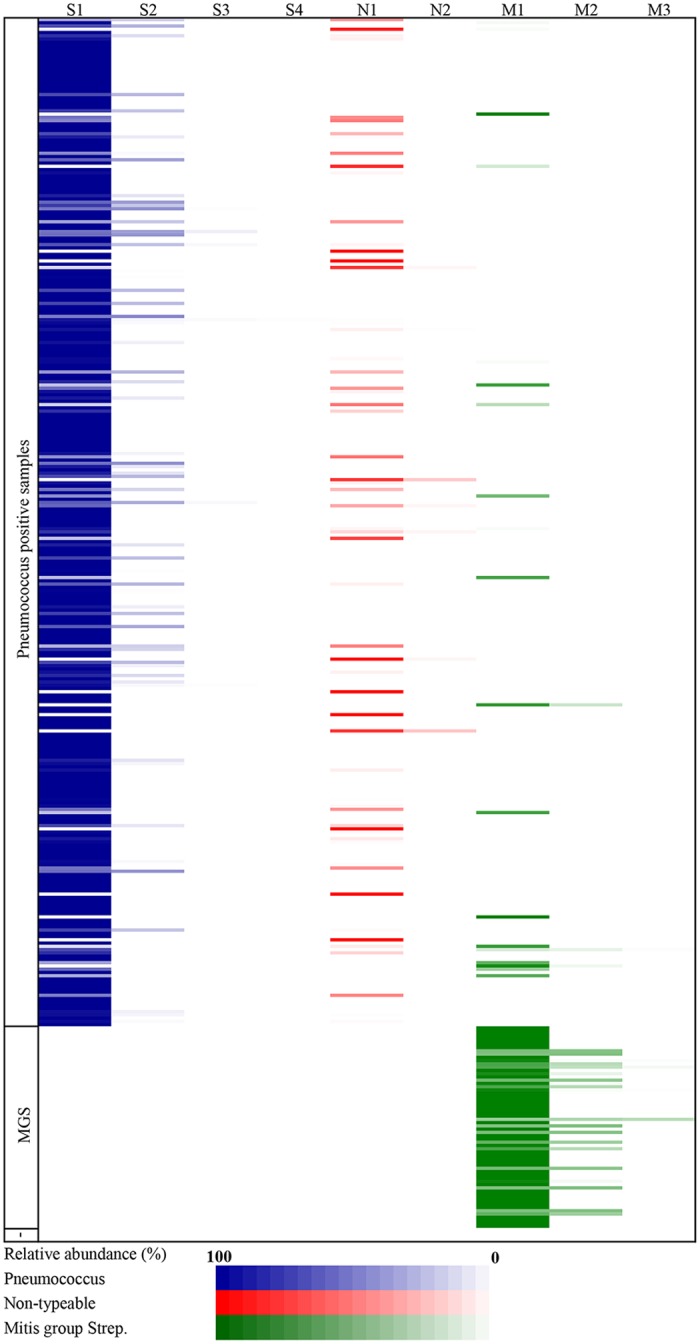
Heat map representation of nasopharyngeal swab isolates from children aged 6 weeks to 24 months from the Kathmandu Valley, Nepal. Isolates are ordered according to participant number and presence of pneumococcus. The depth of colour is representative of the relative abundance of the isolate identified by microarray. Each isolate was divided into three categories: S—Typeable pneumococci, N– Non-typeable pneumococci and, M—Mitis-group Streptococcus. Subsequent isolates within these categories were then ranked 1–4 according to relative abundance.

In addition to typeable pneumococci, the microarray detected non-typeable pneumococci and closely related Mitis-group Streptococci. (MGS) (Fig. 3). Within the pneumococcus positive samples presumptive (as the microarray signature used to differentiate a MGS from a pneumococcus becomes masked when a typeable pneumococcus is already present in high abundance) non-typeable pneumococci were identified 60/309 (19·4%) and MGS 21/309 (6·8%) occasions. Greater than one species of streptococcus were identified on the pneumococcus positive samples 109/309 (35·2%) occasions. From the samples that did not have any pneumococcus identified, 62/66 (93·9%) had a closely related MGS found. These MGS only isolates accounted for 16·1%, 9·4%, and 5·5% of the total swabs in the 6 week to < 6 months, 6 months to < 12 months and 12 to 24 months age groups respectively (p = 0.0022).

### Carriage of Multiple *Streptococcus* spp, Their Relative Abundance, and Inter-serotype Relationships

**Fig 4:**
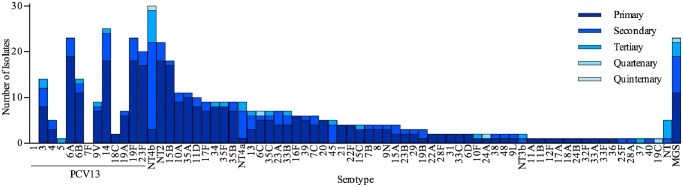
Serotype-specific ranking of multiple pneumococcal carriage. Nasopharyngeal swabs collected from all children aged 6 weeks to 24 months from the Kathmandu Valley, Nepal were analysed by microarray, with each *Streptococcus* isolate from pneumococcus positive swabs ranked according to its relative abundance to other isolates present on the swab.

**Fig 5:**
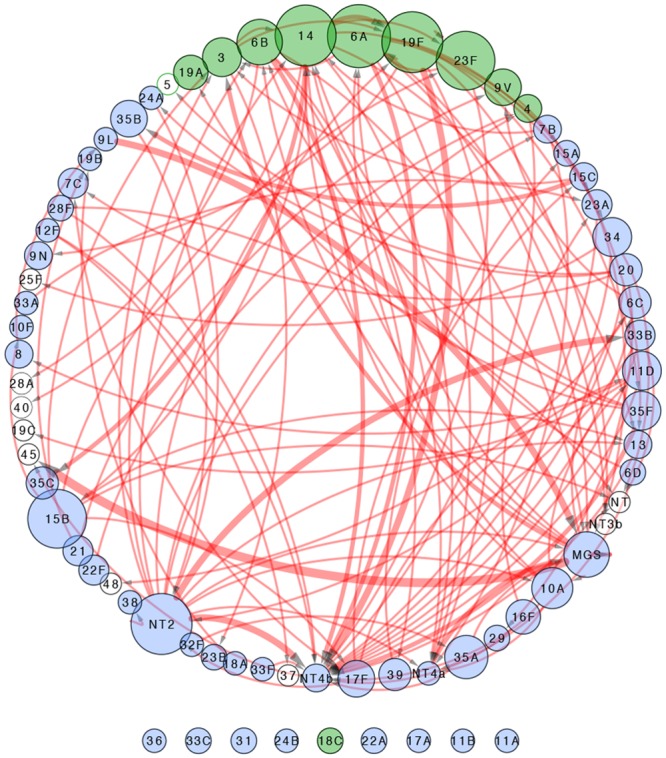
Directional node plot of nasopharyngeal swab pneumococcal serotypes identified by microarray from children aged 6 weeks to 24 months from the Kathmandu Valley, Nepal. The size of each node is representative of the number of primary isolates identified for each serotype. The width of the connecting line is representative of the number of times the connected serotype was found in conjunction with the primary isolate. Green coloured nodes are those serotypes covered by PCV13. Unfilled circles are serotypes that were only found as non-primary isolates.

**Fig 6:**
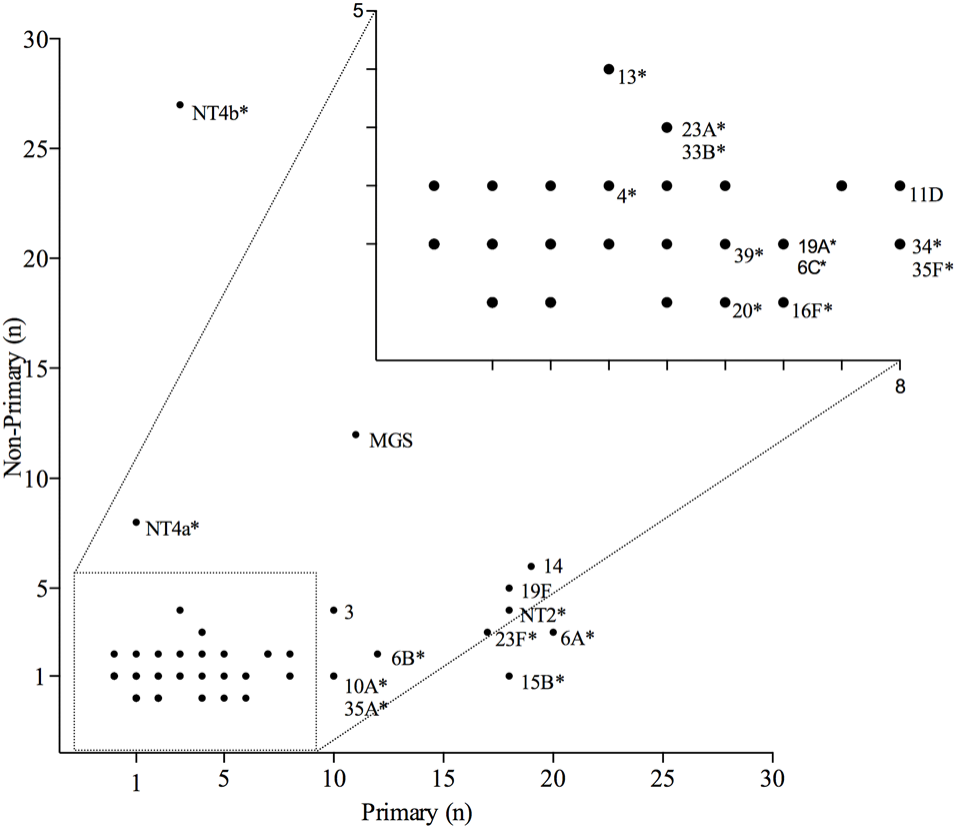
Serotype-specific propensity for isolation as a primary or non-primary isolate. Pneumococcal serotypes identified from nasopharyngeal swabs of Nepalese children aged 6 weeks to 24 months from the Kathmandu Valley, Nepal, were classified as to whether they occurred as a primary or non-primary isolate (*p<0·05). Specifically for each serotype: 15B, 10A, 35A, 34, 35F, 16F, 20, NT4b, 13, and NT4a p<0.0001; 6A p = 0.0005; 6B, 19A and 6C p = 0.0012; 23A and 33B p = 0.0016; NT2 p = 0.0257; 23F p = 0.0035; 4 p = 0.0101. The serotypes, 9V, 14, 19F, 3, 11D, 17F, 35B, 35C, 39, 7C, 45, 15, 7B, 8, 9N, 18C, 15A, 23B, 29, 22A, 28F, 31, 33C, 6D, 19B, 10F, 24A, 38, 48, 9L, 11A, 11B, 12F, 17A, 18A, 24B, 32F, 33A, 33F, 36, 1, 5, 7F, NT3b, 25F, 28A, 37, 40, 19C, and NT were not labelled and/or had non-significant p-values and/or were isolated on less than five occasions. MGS = Mitis-group Streptococcus.

*Streptococcus* spp. identified on each pneumococcal positive swab was ranked according to their measured relative abundance (Fig. 4). A total of 64 different pneumococcal serotypes were identified in addition to MGS and non-typeable pneumococci. Non-typeable pneumococci were classified into four genetically distinct categories (NT2, NT3b, NT4a and NT4b) [26] and the remainder into a non-distinct group (NT). These categories refer to known non-encapsulated lineages that lack *cps* genes. NT4b was most frequently found overall (9·7%) with serotypes 14 (8·1%), 6A (7·4%), 19F (7·4%) and MGS (7·4%) being the next most frequently found. Non-typeable pneumococci NT4b and NT4a were most frequently isolated as secondary or subsequent colonisers (27/30 and 8/9 occasions respectively). PCV13 serotypes represented 143/455 (31·4%) of the total isolates identified on pneumococcus swabs. There were 9 serotypes that were only identified as primary isolates (Figs. 4 & 5). NT2 was most frequently found as a primary isolate with co-colonising pneumococci of other serotypes (17/22 occasions). The relationship between whether a given serotype is more prone to being found as a primary or non-primary isolate is demonstrated in Fig. 6. Serotypes, 10A, 15B, 16F, 20 34, 35A and 35F were highly likely to be identified as primary as opposed to non-primary isolates (p<0·0001) whereas 13, NT4b and NT4a were significantly more likely to be identified as a non-primary isolate (p<0·0001).

### Presence of Antibiotic Resistance Genes

Antibiotic resistance genes within plate sweep samples were detected by microarray analysis. Of those samples that had pneumococcus detected there were 39/76 (51·3%), 64/128 (50%) and 59/105 (56·2%), which contained an antibiotic resistance locus in the 6 week to < 6 months, 6 to < 12 months, and 12 to 24 months age groups respectively (table 2). The ribosomal protection protein encoded by *tetM*, which confers resistance to tetracyclines was the most prevalent locus found in all age groups, followed by *mefA*, encoding a macrolide efflux pump conferring resistance to macrolides. Within the pneumococcal samples that had an antibiotic resistance locus detected 33/162 (20·4%) also had a co-colonising organism (MGS, *Staphylococcus aureus*, *Haemophilus influenzae*, *Moraxella catarrhalis*, *Neisseria meningitidis*) present.

**Table 2:** Presence of Antibiotic Resistance Genes in Pneumococcal Positive Array Samples.

		**6wk-<6mo n = 199**	**6mo-<12mo n = 202**	**12mo-24mo n = 199**	
Total SPN positive subjects (%)		76 (38·2)	128 (63·4)	105 (52·8)	
Total number of SPN positive subjects with an AbR gene (%)		39 (51·3)	64 (50)	59 (56·2)	
Gene, n (% of SPN positive subjects)	*cat*	4 (5·26)	3 (2·34)	3 (2·86)	
	*ermB*	8 (10·5)	12 (9·3)	12 (11·4)	
	*tetM*	26 (34.2)	57 (44·5)	54 (51·4)	p = 0·07
	*aphA3*	3 (4)	1 (1)	1 (1)	
	*sat4*	3 (4)	1 (1)	1 (1)	
	*mefA*	18 (23·7)	27 (21·1)	19 (18·1)	p = 0·65
	*ermC*	4 (5·3)	1 (1)	3 (2·9)	
	*tetK*	1 (1·3)	1 (1)	1 (1)	
	*tetO*	0 (0)	2 (1·6)	0 (0)	

*cat*—chloramphenicol acetyltransferase, *ermB*—rRNA adenine N-6-methyltransferase (resistance to erythromycin), *tetM*—Ribosomal protection protein (conferring resistance to tetracycline), *aphA3*—kanamycin resistance, *sat4*—streptothricin, *mefA*—Macrolide-Lincosamide-Streptogramin B efflux pump, *ermC*—rRNA adenine N-6-methyltransferase (conferring resistance to erythromycin), *tetK*—tetracycline efflux pump, *tetO*—Ribosomal protection protein (conferring resistance to tetracycline).

## Discussion

This is the first study to demonstrate the nasopharyngeal carriage of pneumococcus in Nepalese children using a molecular serotyping method to facilitate the detection of multi-serotype carriage. Overall PCV13 serotypes make up a substantial proportion of those that are detected and concurrent carriage of multiple serotype of pneumococcus is frequent, occurring in 22% of all children studied. The data also indicate the existence of interactions between *Streptococus* spp. that may both facilitate or inhibit co-colonisation. Genes conferring resistance to tetracyclines and macrolides were the most frequently identified antibiotic resistance loci identified from the pneumococcal swabs.

Prior pneumococcal carriage studies performed in children from Nepal have demonstrated overall carriage prevalence’s ranging from 71–88% [27–29]. However two of these studies were in rural populations, whilst the third was in a relatively small cohort of children from an orphanage in Kathmandu. Another study conducted in Patan Hospital, utilising a PCR method for serotyping, demonstrated a carriage prevalence of 58·7% in children aged 6 weeks to 24 months (unpublished data). The carriage prevalence of 51·5% seen in this study, utilising the microarray technique, is broadly in keeping with these prior findings.

Over the last 80 years there have only been a small number of studies looking at the rates of multiple colonisation with pneumococcus, demonstrating a wide range, in different geographical settings, and contexts [15]. From the recent studies, conducted since 1996, the lowest prevalence appears in a study performed on swabs from South African and Israeli children where only 1·5% of all samples collected had more than one pneumococcal isolate [13]. Those recent studies that have been conducted in high carriage (70–92%), resource poor settings, demonstrate 5.1%–24% of pneumococcus positive samples contain more than one serotype [7, 11, 12, 30–33]. Notably the highest prevalence of multiple carriage reported (24%) by Obaro *et al*, in a combination of vaccinated and unvaccinated children, was in the context of the highest rate (92%), and thus presumably the highest density, of overall pneumococcal carriage. These findings are in keeping with the 20·2% prevalence of multiple pneumococcal serotype carriage seen in this present study. However this study provides added depth to this observation, with the inclusion of the non-typeable pneumococci and MGS identified on pneumococcal swabs demonstrating a multiple Streptococcal carriage prevalence of 35·3%.

As in this study, non-typeable pneumococci are the most commonly isolated organisms in other settings throughout Asia [7, 8, 27]. Our data adds to this observation, the fact that that almost all non-typeables isolated were co-colonising isolates. This suggests that there may be synergistic relationships between certain serotypes and non-typeable pneumococci. Capsule formation provides evasion from the host during planktonic growth, however during adherent growth pneumococci have a tendency towards biofilm formation (providing protection from mucosal defences) and down regulation of the capsular gene (*cpsA*) [34]. It may be hypothesised that non-typeable (hence non-capsular) pneumococci may be better suited to adherent growth with biofilm formation within which other co-colonising pneumococci may exist. Interestingly this study demonstrated a significant decrease in carriage of MGS with increasing age and may imply that there is progressive displacement of MGS from the nasopharyngeal niche by other organisms such biofilm forming pneumococci.

In countries where PCVs have been introduced, a reduction in pneumococcal disease and carriage of the vaccine serotypes, with a concurrent increase in non-vaccine serotypes has been observed [20, 30, 35, 36]. It has been suggested that this may be due to increased acquisition of non-vaccine serotypes or perhaps an ‘unmasking’ of serotypes already carried at low burden [12]. Limited data, from a resource poor setting, looking at the impact of PCVs on multiple pneumococcal carriage suggests no difference observed in the prevalence of co-colonisation before and following routine use of PCV7 in infants [10, 31]. The findings from this present study suggest that vaccine introduction into this population will result in replacement of vaccine serotypes with non-vaccine types, most of which will be non-typeable (unencapsulated) pneumococci that are suited to adherent growth and colonisation and have an inherently low invasive disease potential.

**Fig 7:**
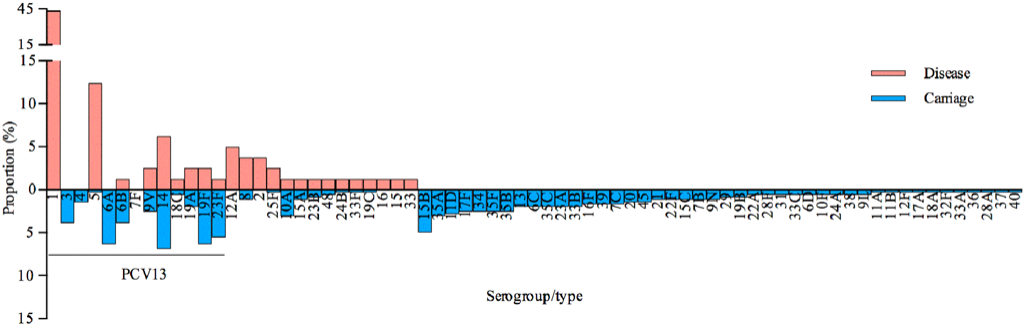
Invasive disease versus carriage mirror plot. The proportion of pneumococcal serotypes isolated by microarray from children aged 6 weeks to 24 months from the Kathmandu Valley, Nepal, compared to the proportion of IPD serotype/groups, identified by PCR, from paediatric in-patients at Patan Hospital, Kathmandu between 2005–2012 (n = 83 cases).

Invasive pneumococcal disease (IPD) isolates from children presenting to the Patan Hospital between 2005 and 2012, were serogroup/typed by PCR (unpublished data) and compared to carriage isolates from this study (Fig. 7). The serotypes covered by PCV13 accounted for 72·8% of IPD isolates with the greatest proportion (43·2%) due to serotype 1. Consistent with previous studies there was a large discordance between carriage and disease for serotypes 1 (0% vs 43·2%) and 5 (0·3% vs 12·4%) which has led to the concept that these are particularly invasive serotypes. Particularly high attack rates have been previously documented in other settings for serotypes 1, 5 and 7F. [4, 5] Notably in this population of Nepalese children no carriage or disease due to serotype 7F was detected. Using these data it can be projected that PCV13 would provide coverage of up to a 72·8% and 42·7% of IPD serotypes and pneumococcal carriage flora respectively.

Pneumococcus frequently undergoes genetic recombination and has a natural competence for DNA uptake [37]. As such the acquisition and dissemination of antibiotic resistance in environments where there is selective pressure due to widespread antibiotic use has been observed [38]. In Nepal there has been widespread use of topical tetracyclines and oral azithromycin in children for the treatment of ocular trachoma, in endemic areas, which has resulted in subsequent resistance being observed in pneumococcal carriage [28]. It is therefore of note that the highest proportions of antibiotic resistance genes detected on pneumococcal swabs (some of which may be carried by MGS) in this study population were also from the tetracycline and macrolide groups.

The limitations of this study are the small age group cohort sizes, cross sectional study design, and in differentiating non-typeable pneumococcus from MGS on pneumococcal positive swabs. A larger cohort size for each of the age groups would have allowed age-specific comparison of pneumococcal carriage prevalence at a serotype level to be performed. Longitudinal data is required to more fully understand the inter-relationships between pneumococcal serotypes in terms of inhibiting or permitting co-colonisation of other serotypes. The microarray signature used to detect MGS may become masked if a typeable pneumococcus is already present at high abundance, whilst one of the genes, *aliB*, used to define non-typeable pneumococcus may also be found in typeable pneumococcus. These inherent difficulties in defining *Streptococcus* spp. even at a high-resolution molecular level, exemplifies the complexity of interactions occurring. The data presented here indicate the need for further work in this population and extension to rural populations among whom there are currently no data on multiple serotype carriage.

The strength of this study is the overall sample size, and sensitivity of the microarray technique for pneumococcal multiple serotype detection which in effect sets the bench mark for all future carriage studies in this region. These data also provide a useful baseline for further surveillance studies in this population with continued disease and carriage monitoring following the implementation of routine immunisation with PCVs which will allow estimates of vaccine efficacy against carriage to be accurately made and may permit inference of effectiveness against disease.

This study has demonstrated that a substantial proportion of Nepalese children from the Kathmandu Valley not only carry pneumococcus but carry more than one serotype. The utilisation of a rapid, less labour intensive, microarray technique has demonstrated the importance of multiple carriage detection and the potential to provide further insight into the bacterial interactions taking place on the nasopharyngeal mucosa. This will allow more accurate measurement of vaccine impact, and offer additional information such as antibiotic resistance and presence of other pathogens. A large fraction of the disease causing serotypes is covered by PCVs and thus lends evidence to the importance of implementing a vaccine within this population. Continued surveillance of nasopharyngeal carriage of multiple serotypes after vaccine implementation in this population will provide a useful insight into, vaccine impact on the circulating pneumococcal reservoir, and the process of serotype (hence phenotypic) and genetic shift.
